# Marine Natural Products as Anticancer Agents

**DOI:** 10.3390/md19080447

**Published:** 2021-08-04

**Authors:** Celso Alves, Marc Diederich

**Affiliations:** 1 MARE—Marine and Environmental Sciences Centre, Polytechnic of Leiria, 2520-630 Peniche, Portugal; 2 Department of Pharmacy, College of Pharmacy, Seoul National University, 1 Gwanak-ro, Gwanak-gu, Seoul 08826, Korea

Cancer remains one of the major threats to human health and one of the deadliest diseases worldwide [1]. Therapy failure and consequent cancer relapse are the main factors contributing to high cancer mortality, making it crucial to find and develop new therapeutic options. Over the last few decades, natural products became one of the key drivers in the drug development of innovative cancer treatments [2]. In opposition to drug development from terrestrial resources, the marine environment only recently emerged as a prolific source of unparalleled structurally active metabolites [3]. Due to their excellent scaffold diversity, structural complexity, and ability to act on multiple cell signaling networks involved in carcinogenesis, marine natural products are ideal candidates to inspire the development of novel anticancer medicines [4,5].

The Special Issue “Marine Natural Products as Anticancer Agents” (https://www.mdpi.com/journal/marinedrugs/special_issues/AnticancerAgents (accessed on 15 June 2021)), gathered nine publications, including a review, a communication, and seven research articles, providing an excellent overview of the chemical richness offered by different marine organisms. Indeed, sponges, myxobacteria, fungi, and soft corals, provide essential scaffolds at the origin of the synthesis and molecular modeling of new anticancer drugs. Marine natural compounds or derived products described in this Special Issue belong to distinct chemical classes, including terpenoids, alkaloids, cyclodepsipeptides, polyketides, and hydroxyphenylacetic acid derivatives. These compounds modulate cancer cell mechanisms in vitro and in vivo in chronic myeloid leukemia, breast adenocarcinoma, prostate carcinoma, and hepatocellular carcinoma cell types. These compounds exhibited high specificity and great affinity to interact with distinct biological targets linked to specific intracellular signaling pathways, including mitochondrial dysfunction, autophagy, endoplasmic reticulum (ER) stress induction, apoptosis, inflammation, migration, and invasion.

Conte and collaborators [6] provide a critical review focused on the ability of marine natural products and their synthetic derivatives to act as epigenetic modulators, discussing advantages, limitations, and potential strategies to improve cancer treatment.

Song and co-workers [7] studied the pharmacological potential of four newly synthesized aplysinopsin derivatives to treat chronic myeloid leukemia, identifying the EE-84 analog as a promising novel drug candidate. EE-84 induces a cytostatic effect, leading to mitochondrial dysfunction, the induction of a senescent-like phenotype, autophagy, and ER stress. Its co-administration with the BH3 mimetic A-1210477 promoted a significant synergistic effect on cancer cell death.

Astaxanthin, a xanthophyll carotenoid that can be found in several marine organisms, including microalgae, bacteria, fungi, sea snails, and sea urchins, among others [8], demonstrated the capacity to decrease the levels of stemness markers (Oct4 and Nanog) in breast carcinoma cells, negatively modulating the expression of pontin and mutant p53 proteins [9]. Furthermore, this carotenoid also inhibited the proliferation of aggressive prostate cancer DU145 cells, triggering apoptotic cell death and decreasing invasion and migration capabilities. From a mechanistic point of view, cell death was triggered by a down-regulation of STAT3 mRNA and protein levels, accompanied by an up-regulation of pro-apoptotic (BAX, caspase3, and caspase9) and a down-regulation of pro-survival (JAK2, BCL-2, and NF-κB) proteins [10]. 

Wu and co-workers [11] studied the therapeutic effects of flaccidoxide-13-acetate diterpenoid, previously isolated from the marine soft coral *Sinularia gibberosa*, on hepatocellular carcinoma (HCC). The authors focused on tumor metastasis formation as this compound decreased the viability, migration, and invasion capability of HCC cells. These effects were mediated by a decrease in the expression levels of matrix metalloproteinases MMP-2, MMP-9, and MMP-13, the suppression of PI3K/Akt/mTOR signaling, the down-regulation of Snail and the up-regulation of E-cadherin protein expression. The modulation of these signaling pathways eventually suppressed the epithelial–mesenchymal transition, invasion, and migration of HCC cells.

Teles and collaborators [12] reported the antitumor properties of a *Penicillium purpurogenum* ethyl acetate extracellular extract, rich in meroterpenoids, using an Erlich mouse model. The extract decreased tumor-associated inflammation and necrosis without causing weight loss nor renal and hepatic toxicity. 

The isolation of new marine natural products with cytotoxic activities was also reported in this Special Issue. Jiang and co-workers [13] described the isolation of three new molecules from *Penicillium chrysogenum* LD-201810, a marine alga-associated fungus: a pentaketide derivative, penilactonol A, and two new hydroxyphenylacetic acid derivatives, (2′R)-stachyline B and (2′R)-westerdijkin A. (2′R)-westerdijkin A was significantly cytotoxic to the hepatocellular carcinoma cell line HepG2.

One of the biggest challenges associated with bioprospecting and the pharmacological application of marine natural products is the low yield of extraction, which can compromise the supply chain to accomplish pre-clinical and clinical trials and thus develop a new drug. Several strategies were developed to overcome these limitations, such as genetic engineering, aquaculture/cultivation, chemical modification, semi-synthesis, and synthesis [14]. Some of those approaches were addressed in this Special Issue. Huang and collaborators [15] reported the isolation of four new biscembranoidal metabolites, sardigitolides A–D (1–4), from the cultured soft coral *Sarcophyton digitatum*. The new biscembranoid, sardigitolide B, significantly reduced the cell viability of breast adenocarcinoma cell lines, MCF-7, and MDA-MB-231.

On the other hand, Zhang and co-workers [16] communicated the optimization of two critical steps in the scale-up synthesis of nannocystin A, a 21-membered cyclodepsipeptide with remarkable anticancer properties, achieving a synthesis at a four hundred milligram scale.

Altogether, the nine publications included in this volume provide an exciting overview of marine natural products as potential therapeutic agents for cancer treatment. 
